# Identification and genetic characterization of bovine hepacivirus in China: A large scale epidemiological study

**DOI:** 10.1016/j.virs.2022.02.003

**Published:** 2022-02-12

**Authors:** Gang Lu, Chaoxi Chen, Ran Shao, Juan Zhang, Jinghao Li, Siqi Cai, Lintao Zhong, Zhiying Lai, Jiajun Ou, Xin Yin, Guihong Zhang, Shoujun Li

**Affiliations:** a College of Veterinary Medicine, South China Agricultural University, Guangzhou, 510642, China; b State Key Laboratory of Veterinary Biotechnology, Harbin Veterinary Research Institute, The Chinese Academy of Agricultural Sciences, Harbin, 150000, China; c Guangdong Provincial Key Laboratory of Prevention and Control for Severe Clinical Animal Diseases, Guangzhou, 510642, China; d Guangdong Technological Engineering Research Center for Pet, Guangzhou, 510642, China; e College of Life Science and Technology, Southwest Minzu University, Chengdu, 610041, China; f General Station of Forest and Grassland Pest Management, National Forestry and Grassland Administration, Shenyang, 110000, China

**Keywords:** Bovine hepacivirus (BovHepV), China, Epidemiology, Genetic diversity, Cattle

## Abstract

Bovine hepacivirus (BovHepV) is a novel virus that was recently discovered in Ghana and Germany in 2015. Until now, this virus has been identified in cattle population worldwide and is classified into subtypes A–G. To fully understand the epidemic situation and genetic characteristic of BovHepV in China, a total of 612 cattle serum samples were collected from 20 farms in seven provinces and municipality in China between 2018 and 2020 and were tested for the presence of BovHepV RNA via semi-nested PCR. The results demonstrated that 49 (8.0%) samples were BovHepV RNA-positive. It is noted that BovHepV infection in yak was confirmed for the first time. BovHepV was detected in all the seven provinces, with the positive rate ranging from 3.1% to 13.3%, which indicates a wide geographical distribution pattern of BovHepV in China. Sequencing results revealed that 5′ UTR of the 49 field BovHepV strains have a nucleotide similarity of 96.3%–100% between each other and 93.9%–100% with previously reported BovHepV strains. In addition, genetic analysis identified five critical nucleotide sites in 5′ UTR to distinguish different subtypes, which was further verified by genomic sequencing and nucleotide similarity analysis. All the 49 Chinese field BovHepV strains were classified into subtype G and this subtype is only determined in cattle in China currently. This study will provide insights for us to better understand the epidemiology and genetic diversity of BovHepV.

## Highlights

• BovHepV was detected in seven provinces in China between 2018 and 2020.
• BovHepV was prevalent in cattle with the positive rate ranging from 3.1% to 13.3%.
• Five critical nucleotide sites in 5′ UTR could distinguish BovHepV subtypes.
• The Chinese field BovHepV strains were classified into subtype G.

## 1 Introduction

Before 2011, hepatitis C virus (HCV) was considered to be the sole member of the genus *Hepacivirus*, family *Flaviviridae*. Recent studies have characterized several novel HCV-related homologs in different animal hosts, including dogs (Kapoor et al., 2011), equines (Burbelo et al., 2012), bats (Quan et al., 2013), rodents (Drexler et al., 2013), monkeys (Lauck et al., 2013), fish (Shi et al., 2016), and cattle (Baechlein et al., 2015; Corman et al., 2015). Currently, a total of fourteen species (*Hepacivirus A–N*) are officially classified in genus *Hepacivirus* by the International Committee on Taxonomy of Viruses (Smith et al., 2016). Bovine hepacivirus (BovHepV), the HCV-related homolog identified in cattle, is the only member of species *Hepacivirus N*.

In 2015, BovHepV was first reported in domestic cattle in Ghana and Germany in two independent studies (Baechlein et al., 2015; Corman et al., 2015). The following epidemiological investigations indicated BovHepV was distributed worldwide, showing no apparent geographic restriction. Until now, BovHepV has been determined in China (Deng et al., 2018; Lu et al., 2018), Brazil (Canal et al., 2017; da Silva et al., 2018), the USA (Sadeghi et al., 2017), Turkey (Yesilbag et al., 2018), and Italy (Elia et al., 2020) as well.

The genome of BovHepV is ∼9*–*10 ​kb in length and has the same genome organization as determined for other viruses in genus *Hepacivirus*, which is composed of a 5′ untranslated region (5′ UTR), a single open reading frame (ORF) encoding a putative polyprotein, and a 3′ UTR (Baechlein et al., 2015; Corman et al., 2015). The BovHepV polyprotein is predicted to cleave into the following ten viral proteins: three putative structural proteins (Core, E1, and E2) and seven putative nonstructural proteins (p7, NS2, NS3, NS4A, NS4B, NS5A, and NS5B).

BovHepV can cause both acute and persistent infections in cattle (Baechlein et al., 2019). Similar to HCV, BovHepV is a hepatotropic virus as highest abundant BovHepV RNA was detected in liver among tissues collected from infected cattle (Baechlein et al., 2019). Previous studies demonstrated HCV-related homolog infection in rats and equines was associated with liver disease in their hosts (Ramsay et al., 2015; Billerbeck et al., 2017). Therefore, BovHepV infection might cause liver damage, possessing a potential threat to bovines’ health.

BovHepV presented highly genetic diversity. Using classification principles for HCV genotypes and subtypes, we previously classified BovHepV strains worldwide into one genotype and seven subtypes (A–F) (Lu et al., 2019). In China, we have determined BovHepV subtype E in dairy cattle in Guangdong Province and detected subtype G in commercial bovine serum samples used for cell culture propagation (Lu et al., 2018, 2019). Notably, BovHepV strains of subtypes E and G have not been identified in cattle outside China.

China owns a large number of cattle. However, knowledge on epidemiology and genetic variability of BovHepV in China is still very limited. In the present study, a screening investigation was systematically performed in the domestic cattle population in seven provinces and municipality to understand the prevalence and genetic diversity of BovHepV circulating in cattle in China.

## 2 Materials and methods

### 2.1 Sample collection

During 2018–2020, a total of 612 serum samples were individually collected from domestic cattle in China. After collection, the samples were immediately transported to laboratory in an icebox and stored at −80 ​°C for further use.

### 2.2 RNA extraction and cDNA synthesis

To investigate the prevalence of BovHepV, total RNA was extracted from 200 ​μL of serum sample from each animal using RNAiso Plus (Takara, China) following the manufacturer's instructions. The resulting RNA was finally diluted in 20 ​μL of nuclease-free water. For cDNA synthesis, 6 ​μL of extracted RNA was used for reverse transcription into cDNA using HiScript 1st Strand cDNA Synthesis Kit (Vazyme, China) with random primers according to the manufacturer's recommendations.

### 2.3 Viral RNA screening

To test the presence of BovHepV RNA, synthesized cDNA was used as template in the following semi-nested PCR as described in our previous report (Lu et al., 2019). Briefly, the PCR reaction system included 2 ​μL of cDNA, 1 ​μL of forward primer, 1 ​μL of reverse primer, 10 ​μL of GenStar Taq Polymerase Premix (Kangrun Chengye, China), and 6 ​μL of nuclease-free water. The applied temperature profiles for both rounds of semi-nested PCR were: 35 cycles of 98 ​°C for 10 ​s, 55 ​°C for 15 ​s, and 72 ​°C for 30 ​s and a final step of 1 cycle at 72 ​°C for 5 ​min. A primer pair of BovHepV3F and BovHepV264R was used in this first round of semi-nested PCR. BovHepV64F and BovHepV264R were used in the second round of PCR. The PCR primers were designed targeting a 201 bp of 5′ UTR region of BovHepV genome. In PCR reaction, water was used as a negative control and a bovine serum sample positive for BovHepV RNA was used as a positive control.

After PCR and 1% gel electrophoresis, agarose gel containing PCR products with the expected size was excised and then purified by gel extraction using universal DNA purification kit (Axygen, the USA). The resulting DNA was sent for sequencing directly from both ends (BGI, China). The sequencing data was subjected to further BLAST analysis in the NCBI database (https://www.ncbi.nlm.nih.gov/).

### 2.4 Viral genome sequencing

To acquire the genome sequence of CQ/166, the genome sequences of BovHepV strains available in the NCBI database were retrieved and then aligned by clusterW method using Bioedit 5.0.7.0. Primer pairs covering BovHepV genome were designed using Oligo 7.0 (Supplementary Table S1). PCR was performed using Phanta Max Super-Fidelity DNA Polymerase (Vazyme, China) and CQ/166 cDNA was used as template. Annealing temperature for each PCR procedure was set at 55 ​°C. The purified blunt-ended PCR products were individually cloned with a pCloneEZ-blunt kit (Clone smarter, the USA) and then transformed into *E. coli* DH5α competent cells (Weidi, China). After verified by PCR, positive bacterial clones were sent for sequencing (BGI, China). After sequencing, the raw data was assembled by SeqMan 7.1.0 and the genome sequence of CQ/166 was obtained.

### 2.5 Phylogenetic analysis

Potential recombination events between BovHepV strains were detected using RDP4 and seven methods were used for the analysis, including RDP, GENECONV, Chimaera, MaxChi, BootScan, SiScan, and 3 Seq.

To determine the evolutionary status of BovHepV strains circulating in domestic cattle in China, a phylogenetic tree was generated to compare the sequences obtained in the present study with previously reported sequences from human and other animals. The sequences were aligned by clusterW method using MegAlign 7.1.0. Two maximum likelihood phylogenetic trees were constructed using MEGA 7.0 based on viral polyprotein coding nucleotide sequences respectively. After estimated by “Find Best DNA/Protein Models”, general time reversible (GTR) with gamma distribution with invariant sites (G ​+ ​I) substitution models were used to generate phylogenetic trees and bootstrapping values were set to 1000.

### 2.6 Nucleotide sequence accession numbers

The sequences of 5′UTR sequences for 48 field BovHepV strains have been deposited in the GenBank database under numbers MW830328–MW830375. The accession number of the genomic sequence of CQ/166 was MW830376. The detailed information on the 49 field strains was listed in Supplementary Table S2.

## 3 Results

### 3.1 Prevalence of BovHepV in China

**Fig. 1:**
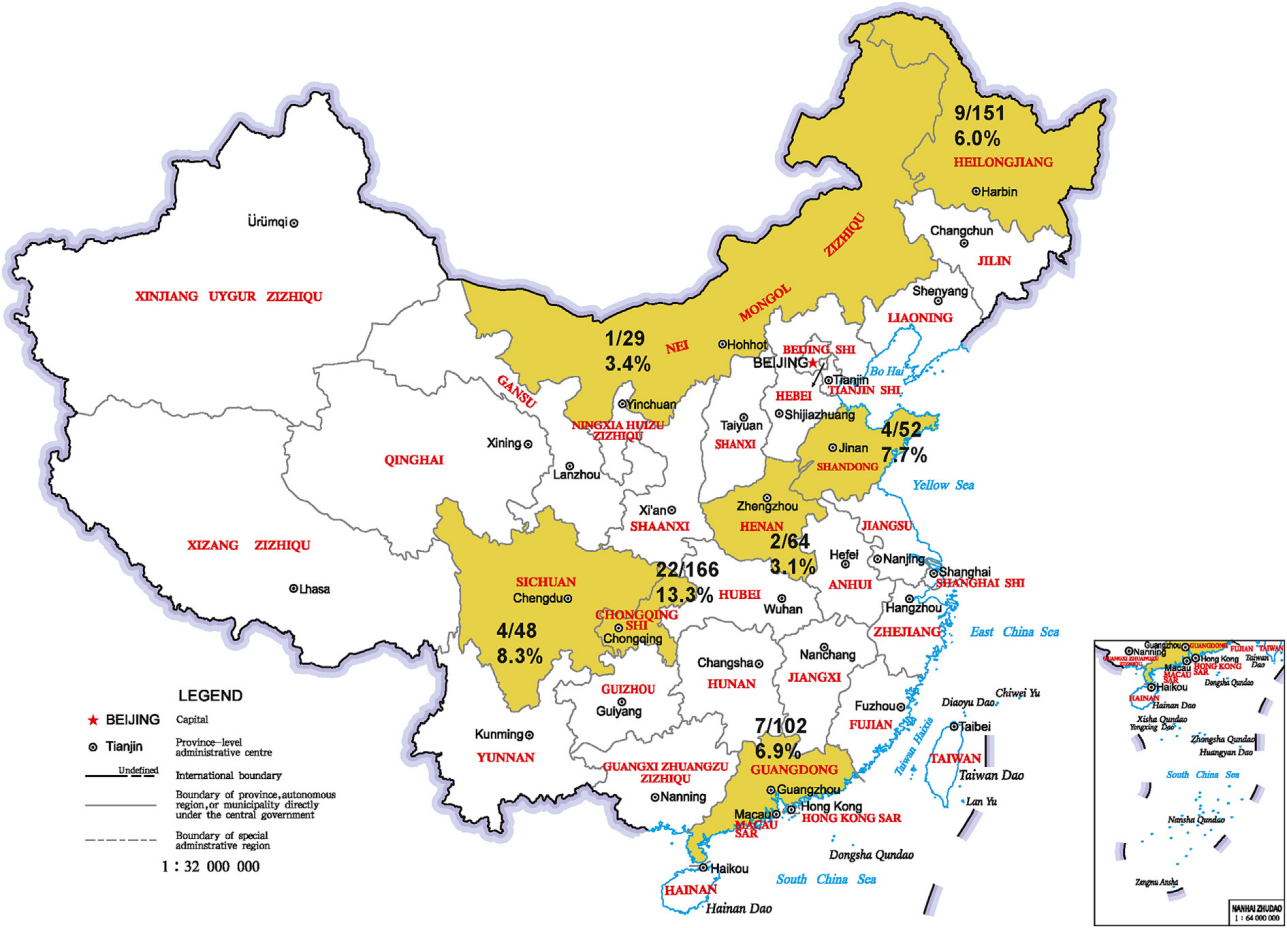
Locations of sample collection. The sampled provinces and municipality are indicated in yellow. The number of BovHepV-RNA positive samples/total tested samples is demonstrated in each province.

A total of 612 serum samples obtained from domestic cattle individuals were involved in BovHepV RNA screening investigations in this study. Samples were collected from seven provinces and municipality in northern, central, and southern China, including Heilongjiang, Inner Mongolia, Shandong, Henan, Sichuan, Chongqing, and Guangdong (Fig. 1). The animals were kept in 20 farms. Among the 612 serum samples, 48 samples were obtained from yak in Sichuan Province. The remaining samples were collected from beef cattle.

As 5′ UTR of BovHepV is relatively conserved, a semi-nested PCR test targeting partial 5′ UTR was used in screening for BovHepV infection in cattle from different provinces. After RNA extraction, cDNA synthesis, PCR and gel electrophoresis, 49 serum samples displayed bands with an expected size. After sequencing and BLAST analysis, it indicated all the 49 PCR-positive serum samples contained BovHepV RNA. Therefore, the positive rate of BovHepV in China during 2018–2020 in the present study was approximately 8.0%. The positive rate for BovHepV infection varied in different provinces, ranging from 3.1% to 13.3% (Fig. 1). The highest and lowest positive rates for BovHepV infection were from sera collected in Chongqing and Henan respectively. It was noted that 4 out of 48 serum samples from yak was BovHepV-positive, indicating BovHepV infection was firstly determined in this unique cattle species.

### 3.2 Genetic analysis of BovHepV in China

After removing the sequences of PCR primers, the partial 5′ UTR of all the 49 Chinese field BovHepV strains determined in the present study had a nucleotide length of 164 bp and was then aligned with that of previously reported 33 BovHepV strains in China, Ghana, Germany, Brazil, and Italy (Supplementary Table S3). Further analysis demonstrated that 5′ UTR of the 49 strains had a nucleotide similarity of 96.3%–100% between each other and had a nucleotide similarity of 96.3%–99.4%, 94.5%–97.6%, 93.9%–96.3%, 95.7%–97.6%, 95.7%–97.6%, 93.9%–97.0%, and 97.6%–100% with 33 BovHepV strains of subtypes A–G respectively (Supplementary Table S3).

**Fig. 2:**
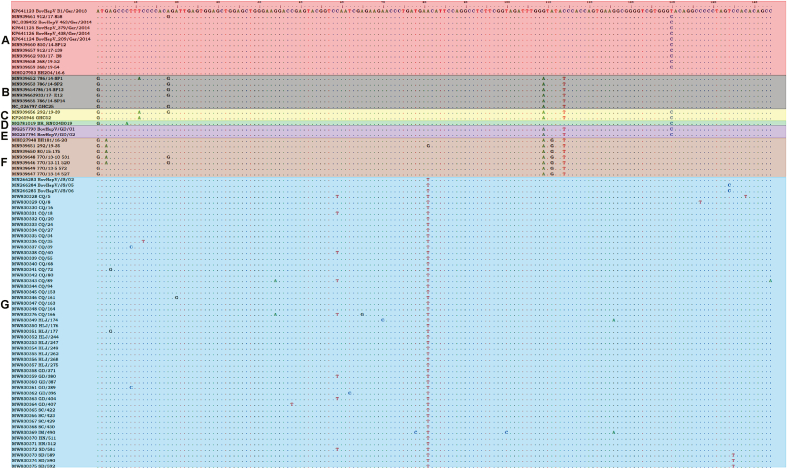
Alignment results of BovHepV 5′ UTR. The alignment result of nucleotide position 84–247 was shown. BovHepV strains of subtypes A–G are indicated by different colors. The five nucleotide positions (84, 164, 192, 194, 197) for subtype classification were indicated by arrow.

The genome sequence of a German BovHepV strain BovHepV_B1/Ger/2013 was used as reference and the nucleotide position of 5′ UTR in other BovHepV strains was numbered according to this strain. A total of 16 unique nucleotide substitutions (A87G, T92C, A103G, C90T, G127A, C131T, T145C, A148G, A153C, T161C, T183C, G209A, C230T, C238T, C241T, C247T) in the 5′ UTR were observed in 21 BovHepV strains sequenced in our study (Fig. 2). The 5′ UTR region of the Chinese BovHepV strains showed genetic diversity.

The alignment results based on 5′ UTR of BovHepV strains demonstrated that five critical nucleotide sites (84, 164, 192, 194, and 197) in 5′ UTR could well differentiate subtypes A–G from each other (Table 1). For subtype G, the special nucleotides at the five sites were A, T, G, A, and C respectively. It is noted that 5′ UTR of all the 49 Chinese field BovHepV strains sequenced in the present study were consistent with characteristics of subtype G. The result indicated BovHepV of subtype G was prevalent in China within a wide geographic area.

**Table 1:** The critical nucleotide sites in BovHepV 5′ UTR that could differentiate subtypes A–G.

BovHepV subtype	Nucleotide position				
	84	164	192	194	197
A	A	A	G	A	C
B	G	A	A	A	T
C	G	A	A	A	T
D	G	A	G	A	C
E	A	A	A	A	T
F	G	A/G^a^	A	G	T
G	A	T	G	A	C

a The nucleotide G at position 164 was only observed in one Italian BovHepV strain 292/19–35.

### 3.3 Genomic sequencing and analysis of CQ/166

From the analysis of 5′ UTR of 49 Chinese BovHepV strains, we found that BovHepV strain CQ/166 detected in Chongqing had the largest number of unique nucleotide substitutions. To further confirm the presence of BovHepV of subtype G in China, the representative strain CQ/166 was chosen to obtain its genome sequence. After PCR and assemble, the near-complete genome sequence of CQ/166 was acquired, including a 236-nucleotide partial 5′ UTR and an 8339-nucleotide complete polyprotein gene. Polyprotein gene of CQ/166 had a nucleotide identity of 92.2%–92.4% and 79.1%–84.6% with that of representative BovHepV strains of subtype G and subtypes A–F respectively (Table 2). Polyprotein of CQ/166 had amino acid similarity at 97.6%–97.7% and 91.7%–95.8% with that of BovHepV strains subtype G and subtypes A–F respectively (Table 2). BovHepV genotypes and subtypes were differentiated between each other by *P*-distance of >0.23 and ​>0.15 ​at amino acid and nucleotide level respectively. Accordingly, CQ/166 was classified into subtype G of genotype 1.

**Table 2:** The nucleotide identity and amino acid similarity of polyprotein between CQ/166 and BovHepV strains of subtypes A–G.

BovHepV subtype	Nucleotide identity (%)	Amino ​acid ​similarity (%)
A	84.1–84.6	95.3–95.8
B	79.7–80.2	92.6–92.9
C	80.4	92.1–92.6
D	82.2–82.5	94.3–94.4
E	79.1–79.2	91.8
F	79.5	91.7
G	92.2–92.4	97.6–97.7

Nineteen representative BovHepV strains of subtype A–G were included in the analysis.

Subtype A: BovHepV_B1/Ger/2013 (KP641123); BovHepV_379/Ger/2014 (KP641125); BovHepV_463/Ger/2014 (KP641127); BovHepV_209/Ger/2014 (KP641124); BovHepV_438/Ger/2014 (KP641126); BH204/166 (MH027953).

Subtype B: GHC100 (KP265950); GHC25 (NC_026797); GHC85 (KP265948).

Subtype C: GHC52 (KP265946); GHC55 (KP265947).

Subtype D: BR_MA236B017 (MG781018); BR_RN034B019 (MG781019).

Subtype E: BovHepV/GD/01 (MG257793); BovHepV/GD/02 (MG257794).

Subtype F: BH181/1620 (MH027948).

Subtype G: BovHepV/JS/02 (MN266283); BovHepV/JS/05 (MN266284); BovHepV/JS/06 (MN266285).

**Fig. 3:**
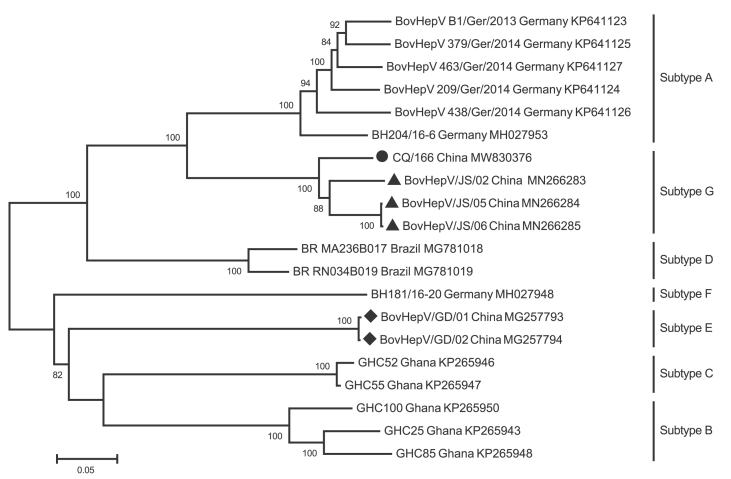
Phylogenetic analysis of CQ/166 based on polyprotein gene. BovHepV strain CQ/166 and the Chinese BovHepV strains of subtype E (BovHepV/GD/01, BovHepV/GD/02) and G (BovHepV/JS/02, BovHepV/JS/05, BovHepV/JS/06) are indicated with circles, diamonds, and triangles respectively.

Potential recombination events between BovHepV strains were detected by bioinformatics methods and no potential natural recombination events were confirmed. Phylogenetic analysis of BovHepV strains based on their polyprotein genes clearly showed that BovHepV strains of the same subtype were clustered together with each other. CQ/166 had a close relationship with three BovHepV strains (BovHepV/JS/02, BovHepV/JS/05, BovHepV/JS/06) that were determined in commercial bovine serum samples for cell culture in China and was grouped into subtype G (Fig. 3).

## 4 Discussion

In 2018, we reported the presence of BovHepV subtype E in 8 out of 102 serum samples that were collected from dairy cattle in one farm in Guangdong Province (Lu et al., 2018). Furthermore, we determined BovHepV subtype G in bovine serum for cell cultures commercially available in Jiangsu Province in central China (Lu et al., 2019). However, the sample size in previous studies is constrained. It is still necessary to fully understand the epidemic situation of this virus in China and the geographical distribution characteristics of each subtype. In this study, we tested BovHepV RNA in 612 serum samples from seven provinces and municipality in China and confirmed the presence of BovHepV in all seven provinces (Fig. 1). The result indicated that BovHepV was prevalent nationwide in China, covering the northern, central and southern areas.

It is noteworthy that only BovHepV subtype G was detected in the present study. The reason is unclear for that BovHepV subtype E was not detected in the serum samples. We used the same primers and PCR procedures as previous studies (Lu et al., 2018), but the sequencing results demonstrated only the positive control contained BovHepV subtype E-RNA and no serum samples in current study were positive for BovHepV subtype E-RNA. It is possible that BovHepV subtype E is no longer prevalent in China. Alternatively, BovHepV of this subtype is circulating at a low prevalence rate. Considering the large number of samples involved in our study, we could conclude that the dominant prevalent BovHepV in China was of subtype G.

Yak (*Bos grunniens*) is a unique cattle species. It lives in the cold plateau around the Qinghai Tibet Plateau in Northwest China. The local altitude is ​> ​3000 ​m and the annual average temperature is ​< ​0 ​°C (Xu et al., 2014). Currently, knowledge on pathogens infecting yak is limited. However, there is increasing evidence that yak can be infected with viruses that are detected in other cattle species, such as Hepatitis E virus and bluetongue virus (Mauroy et al., 2008; Xu et al., 2014). Our present study provides the first evidence that yak could be infected with BovHepV, expanding the known virus spectrum that can infect this animal.

The principle that was used to distinguish different BovHepV subtypes is to estimate *P*-distance of polyprotein gene at nucleotide level (da Silva et al., 2018; Lu et al., 2019). Polyprotein gene of BovHepV is ∼8.3 ​kb in length, covering ∼80% of the total nucleotide sequence in the whole viral genome. Obtaining the long sequences of polyprotein gene is time-consuming, which becomes particularly difficult when handling samples with low quality and/or containing low viral loads. In our study, the partial BovHepV 5′ UTR with a nucleotide length of 164 bp was acquired by semi-nested PCR. The genome sequences of all known BovHepV strains with a definite subtype were retrieved and aligned. It was found that mutations at five critical nucleotide sites in the 5′ UTR region were sufficient to distinguish different subtypes of the virus, which was further verified by genomic sequencing and nucleotide similarity analysis on BovHepV strains detected in the present study. This provided an alternative strategy to differentiate BovHepV subtypes.

## 5 Conclusions

In this study, we collected 612 cattle serum samples from seven provinces and municipality in China and identified 49 samples containing BovHepV RNA, indicating BovHepV was widely spread in China. We sequenced partial 5′ UTR of those 49 field BovHepV strains and found critical nucleotide sites in 5′ UTR to distinguish different subtypes of the virus. These results of this study will enable a better understanding of prevalence and genetic variability of BovHepV in China.

## Data availability

The sequences of 5′UTR sequences for 48 field BovHepV strains have been deposited in the GenBank database under numbers MW830328–MW830375. The accession number of the genomic sequence of CQ/166 was MW830376.

## Ethical statement

All institutional and national guidelines for the care and use of laboratory animals were followed. All procedures in the animal experiments met the requirements and were approved by the Experimental Animal Welfare Ethics Committee of the South China Agricultural University.

## Author contributions

Gang Lu: investigation, funding acquisition, project administration, data curation, writing – original draft, writing – review & editing. Chaoxi Chen: conceptualization, methodology, resources, software, validation, visualization. Ran Shao: methodology, resources, software, validation, visualization. Juan Zhang: methodology, resources, software, validation, visualization. Jinghao Li: investigation, methodology. Siqi Cai: investigation, methodology. Lintao Zhong: investigation, methodology. Zhiying Lai: investigation, methodology. Jiajun Ou: investigation, methodology. Xin Yin: funding acquisition, investigation, supervision, validation, visualization, writing – review & editing. Guihong Zhang: funding acquisition, investigation, methodology, supervision, validation, visualization. Shoujun Li: funding acquisition, investigation, methodology, supervision, validation, visualization.

## Conflict of interest

The authors declare that they have no conflict of interest.
