# Supplementary material for: Identification and genetic characterization of bovine hepacivirus in China: A large scale epidemiological study
Source: Virol Sin. 2022 Feb 12;37(2):223–8. doi: 10.1016/j.virs.2022.02.003 (PMC9170970; doi:10.1016/j.virs.2022.02.003)
Supplement: Multimedia component 1 [file mmc1.docx]

***Virologica Sinica***

**Supplementary Data**

**Identification and genetic characterization of Bovine Hepacivirus in China: A large scale epidemiological study**

Gang Lu ^a, b, c, d, 1^, Chaoxi Chen ^e, 1^, Ran Shao ^a, c, d^, Juan Zhang ^b^, Jinghao Li ^f^, Siqi Cai ^a, c, d^, Lintao Zhong ^a, c, d^, Zhiying Lai ^a, c, d^, Jiajun Ou ^a, c, d^, Xin Yin ^b, **^, Guihong Zhang ^a, *^, Shoujun Li ^a, c, d *^

^a^ College of Veterinary Medicine, South China Agricultural University, Guangzhou 510642, China

^b^ State Key Laboratory of Veterinary Biotechnology, Harbin Veterinary Research Institute, the Chinese Academy of Agricultural Sciences, Harbin 150000, China.

^c^ Guangdong Provincial Key Laboratory of Prevention and Control for Severe Clinical Animal Diseases, Guangzhou 510642, China

^d^ Guangdong Technological Engineering Research Center for Pet, Guangzhou 510642, China

^e^ College of Life Science and Technology, Southwest Minzu University, Chengdu 610041, China.

^f^ General Station of Forest and Grassland Pest Management, National Forestry and Grassland Administration, Shenyang 110000, China.

^1^ Gang Lu and Chaoxi Chen contributed equally to this work.

*Correspondence authors

Email address:

[shoujunli@scau.edu.cn](mailto:shoujunli@scau.edu.cn) (S. Li); guihongzh@scau.edu.cn (G. Zhang); yinxin@caas.cn (X. Yin).

ORCID: 0000-0002-9560-2826 (S. Li); 0000-0003-1236-1850 (G. Zhang); 0000-0003-2357-6718 (X. Yin).

**Table S1 Primers used for genomic sequencing of CQ/166.**

| Primer name | Primer sequences (5′→3′) | Product size (bp) |
| --- | --- | --- |
| BovHepV-59F ^a^ | GCCCCTAGTAGGAGGCG | 1025 |
| BovHepV-1059R ^b^ | ATTAAGGGTAAGATGAGTAGGTAGA |  |
| BovHepV-800F ^a^ | ATTGTTGTCAAGATGATGATGTCCT | 760 |
| BovHepV-1537R ^b^ | AGGCCAGGAAGGTAAGGCAGCCG |  |
| BovHepV-1512F ^a^ | CGACGGCAAAAATGTGTCCTTAG | 1644 |
| BovHepV-3133R ^b^ | TCATCAAACACCACAGGTGGGAG |  |
| BovHepV-3003F ^a^ | ACTTTGGGAGGGCAATATTTTCA | 1594 |
| BovHepV-4574R ^b^ | CCACGTAAAGGAAGTGGAATGTA |  |
| BovHepV-4320F ^a^ | GGGAGCTCCTGGTGTGTACTATT | 1264 |
| BovHepV-5559R ^b^ | AGGAGAGATGACATTGGATTTAGCA |  |
| BovHepV-5444F ^a^ | TAAATTGTGCCTTTGCCCCGG | 1498 |
| BovHepV-6919R ^b^ | GCAAGATGGGCTACATAAGGGAG |  |
| BovHepV-68541F ^a^ | AAGATTCCCTTTCCGCTTTGG | 1441 |
| BovHepV-8271R ^b^ | AATCCGTCGTTTCCATGCATG |  |
| BovHepV-8025F ^a^ | TTTTTTCTTGACGCGTGACCC | 610 |
| BovHepV-8610R ^b^ | TCAGTGTTTGAGGAGGAAAAACAGT |  |

^a^ Forward; ^b^ Reverse.

Primers are designed based on the genomic sequence of BovHepV_209/Ger/2014 (KP641124).

**Table S2 Detailed information on 49 field BovHepV strains determined in the present study.**

| Strain name | Sampling time | Sampling province | Accession number |
| --- | --- | --- | --- |
| CQ/5 | 2018 | Chongqing | MW830328 |
| CQ/8 | 2018 | Chongqing | MW830329 |
| CQ/16 | 2018 | Chongqing | MW830330 |
| CQ/18 | 2018 | Chongqing | MW830331 |
| CQ/20 | 2018 | Chongqing | MW830332 |
| CQ/24 | 2018 | Chongqing | MW830333 |
| CQ/27 | 2018 | Chongqing | MW830334 |
| CQ/34 | 2018 | Chongqing | MW830335 |
| CQ/35 | 2018 | Chongqing | MW830336 |
| CQ/39 | 2018 | Chongqing | MW830337 |
| CQ/40 | 2018 | Chongqing | MW830338 |
| CQ/55 | 2018 | Chongqing | MW830339 |
| CQ/68 | 2018 | Chongqing | MW830340 |
| CQ/72 | 2018 | Chongqing | MW830341 |
| CQ/80 | 2018 | Chongqing | MW830342 |
| CQ/89 | 2018 | Chongqing | MW830343 |
| CQ/94 | 2018 | Chongqing | MW830344 |
| CQ/153 | 2018 | Chongqing | MW830345 |
| CQ/161 | 2018 | Chongqing | MW830346 |
| CQ/163 | 2018 | Chongqing | MW830347 |
| CQ/164 | 2018 | Chongqing | MW830348 |
| CQ/166 | 2018 | Chongqing | MW830376 |
| HLJ/174 | 2018 | Heilongjiang | MW830349 |
| HLJ/176 | 2018 | Heilongjiang | MW830350 |
| HLJ/177 | 2018 | Heilongjiang | MW830351 |
| HLJ/244 | 2018 | Heilongjiang | MW830352 |
| HLJ/247 | 2018 | Heilongjiang | MW830353 |
| HLJ/249 | 2018 | Heilongjiang | MW830354 |
| HLJ/262 | 2018 | Heilongjiang | MW830355 |
| HLJ/268 | 2018 | Heilongjiang | MW830356 |
| HLJ/275 | 2018 | Heilongjiang | MW830357 |
| GD/371 | 2020 | Guangdong | MW830358 |
| GD/380 | 2020 | Guangdong | MW830359 |
| GD/387 | 2020 | Guangdong | MW830360 |
| GD/389 | 2020 | Guangdong | MW830361 |
| GD/396 | 2020 | Guangdong | MW830362 |
| GD/404 | 2020 | Guangdong | MW830363 |
| GD/407 | 2020 | Guangdong | MW830364 |
| SC/422 | 2018 | Sichuan | MW830365 |
| SC/423 | 2018 | Sichuan | MW830366 |
| SC/429 | 2018 | Sichuan | MW830367 |
| SC/430 | 2018 | Sichuan | MW830368 |
| IM/490 | 2019 | Inner Mongolia | MW830369 |
| HN/511 | 2019 | Henan | MW830370 |
| HN/512 | 2019 | Henan | MW830371 |
| SD/581 | 2020 | Shandong | MW830372 |
| SD/589 | 2020 | Shandong | MW830373 |
| SD/590 | 2020 | Shandong | MW830374 |
| SD/592 | 2020 | Shandong | MW830375 |

**Table S3 Detailed information on 33 previously reported BovHepV strains in China, Ghana, Germany, Brazil, and Italy.**

| **Accession number** | **Strain name** | **Genotype** | **Country** | **Nucleotide similarity (%)** |
| --- | --- | --- | --- | --- |
| KP641123 | BovHepV B1/Ger/2013 | A | Germany | 97.6–99.4 |
| MN939661 | 912/17-858 | A | Italy | 96.3–98.2 |
| NC_038432 | BovHepV 463/Ger/2014 | A | Germany | 97.0–98.8 |
| KP641125 | BovHepV_379/Ger/2014 | A | Germany | 97.0–98.8 |
| KP641126 | BovHepV_438/Ger/2014 | A | Germany | 97.0–98.8 |
| KP641124 | BovHepV_209/Ger/2014 | A | Germany | 97.0–98.8 |
| MN939660 | 850/14-SP12 | A | Italy | 97.0–98.8 |
| MN939657 | 912/17-139 | A | Italy | 97.0–98.8 |
| MN939662 | 933/17- B8 | A | Italy | 97.0–98.8 |
| MN939658 | 368/19-52 | A | Italy | 97.0–98.8 |
| MN939659 | 368/19-54 | A | Italy | 97.0–98.8 |
| MH027953 | BH204/16-6 | A | Germany | 97.6–99.4 |
| MN939652 | 786/14-SP1 | B | Italy | 94.5–96.3 |
| MN939653 | 786/14-SP2 | B | Italy | 95.1–97.0 |
| MN939654 | 786/14-SP13 | B | Italy | 95.1–97.0 |
| MN939663 | 933/17- E12 | B | Italy | 95.1–97.0 |
| MN939655 | 786/14-SP14 | B | Italy | 95.7–97.6 |
| NC_026797 | GHC25 | B | Ghana | 95.1–97.0 |
| MN939656 | 292/19-39 | C | Italy | 93.9–95.7 |
| KP265946 | GHC52 | C | Ghana | 94.5–96.3 |
| MG781019 | BR_RN034B019 | D | Brazil | 95.7–97.6 |
| MG257793 | BovHepV/GD/01 | E | China | 95.7–97.6 |
| MG257794 | BovHepV/GD/02 | E | China | 95.7–97.6 |
| MH027948 | BH181/16-20 | F | Germany | 94.5–96.3 |
| MN939651 | 292/19-35 | F | Italy | 94.5–96.3 |
| MN939650 | 80/15-175 | F | Italy | 94.5–96.3 |
| MN939648 | 770/13-10 531 | F | Italy | 93.9–95.7 |
| MN939646 | 770/13-11 520 | F | Italy | 93.9–95.7 |
| MN939649 | 770/13-5 572 | F | Italy | 95.1–97.0 |
| MN939647 | 770/13-14 527 | F | Italy | 95.1–97.0 |
| MN266283 | BovHepV/JS/02 | G | China | 98.2–100 |
| MN266284 | BovHepV/JS/05 | G | China | 97.6–99.4 |
| MN266285 | BovHepV/JS/06 | G | China | 97.6–99.4 |

The 5′ UTR sequences of 33 previously reported BovHepV strains in China, Ghana, Germany, Brazil, and Italy were aligned with that of 49 field BovHepV strains determined in the present study. The nucleotide similarity between the 49 field BovHepV strains and each of the 33 previously reported BovHepV strains was indicated.
